# Pipkin type III femoral head fracture–dislocation combined with complicated acetabular fracture: A rare case report and literature review

**DOI:** 10.1097/MD.0000000000009214

**Published:** 2017-12-15

**Authors:** Bei Zhao, Hao Li, Jun Yan, Li-Ren Han, Xiao-Fei Yang

**Affiliations:** Department of Orthopaedics, Liaocheng People's Hospital and Liaocheng Clinical School of Taishan Medical University, Liaocheng, Shandong, China.

**Keywords:** acetabular fracture, case report, femoral neck fracture, Pipkin fracture

## Abstract

**Rationale:**

Pipkin III fracture, which is characterized by high risk of avascular necrosis of the femoral head, is extremely rare. It is more difficult to treat and has a worse prognosis when accompanied with severe acetabular fractures. Few studies show that both Pipkin type III femoral head fracture–dislocation and complicated acetabular fracture presented in one patient.

**Patient concerns:**

A 34-year-old male suffered a terrible traffic accident with a serious damage to the left side when he was sitting in the car's cockpit. Pelvic radiograph and 3-dimensional reconstruction of computed tomography revealed characteristics of fractures before the emergency operation.

**Diagnosis:**

Pipkin III fractures combined with complicated acetabular fracture.

**Interventions:**

Firstly, we used combined anterior and posterior approach for treatment to fix the femoral head fractures. Then, we completed anatomical reduction of fractures with countersunk head screw, hollow screw, and reconstruction plate.

**Outcomes:**

At the 12-months follow-up, the patient could walk freely and perform activities of daily living without necrosis of femoral head and heterotopic ossification.

**Lessons:**

Although there are serious complications in Pipkin III fractures combined with complicated acetabular fracture, early surgical treatment with appropriate approach and fixation could get satisfactory results.

## 1 Introduction

With high energy injuries increasing, hip complicated fractures are frequent and the treatment of injuries depends on the age of patient and the extent of injury. Fracture of the femoral head was first reported by Birkett in 1869.^[1]^ Pipkin divided Epstein–Thomas type V fracture dislocation into 4 subtypes according to the type of fracture in 1957.^[2]^ According to the classification, only Pipkin type III injuries contain fracture of femoral neck. As the complexity is difficult to reconstruct into the natural hip joint, type III injuries have worse functional outcomes in contrast to the relative simpler injuries of Pipkin type I or II.^[3]^ To the best of our knowledge, few report on Pipkin type III femoral head fracture–dislocation combined with complicated acetabular fracture exists in literatures. We operated on a 34-year-old male who suffered from such a serious injury. After a year follow-up time, the patient showed excellent clinical function and remained satisfied with the surgical outcome.

## 2 Consent

The current study was approved by ethics committee of the Liaocheng People's Hospital and Liaocheng Clinical School of Taishan Medical University. There is no need to obtain informed consent from the patient because all the data were collected and analyzed anonymously.

## 3 Case report

**Figure 1:**
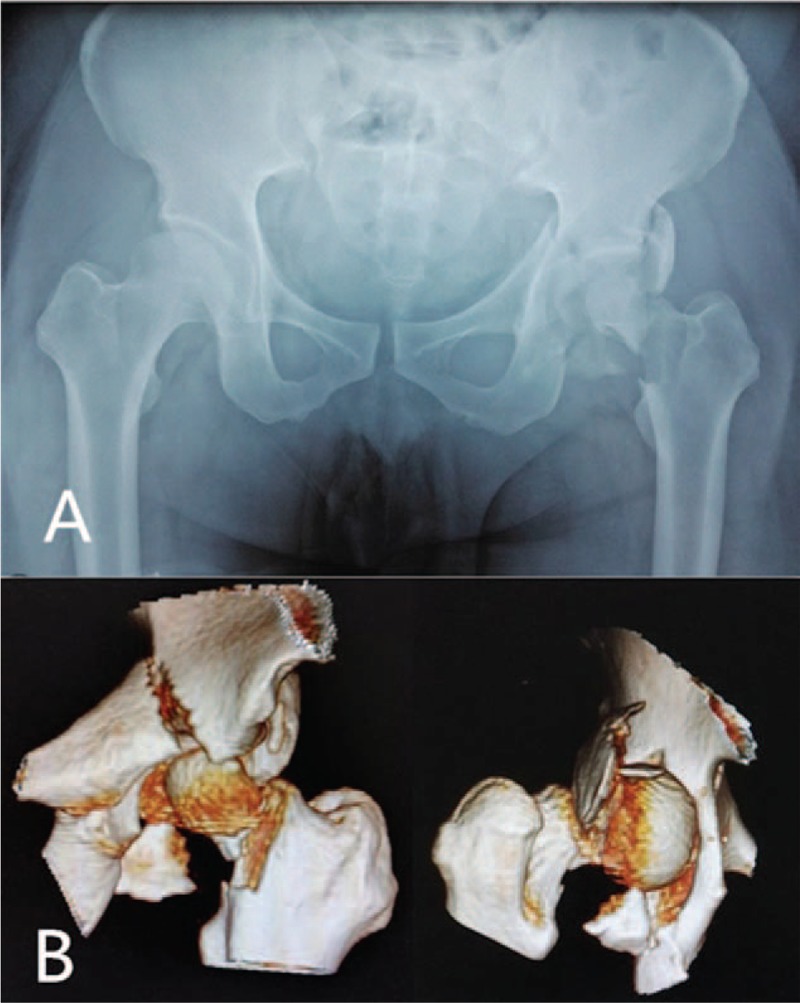
(A) Anteroposterior x-ray image of the pelvis. (B) Anteroposterior three-dimensional computed tomography of hip showing femoral head, femoral neck and transverse acetabular fractures. Posteroanterior three-dimensional computed tomography showing fractures of the femoral neck and posterior wall of acetabulum.

A 34-year-old male suffered a terrible traffic accident with a serious damage to the left side when he was sitting in the car's cockpit. The patient was given x-ray and computed tomography (CT) examination in the emergency. The imaging revealed the transverse-posterior wall acetabular fracture with an associated femoral head and femoral neck fracture (Fig. 1A and B). Because of severe fractures and dislocation, femoral head was blocked by the posterior margin of acetabulum. After recognizing the irreducibility and severity, we opted for emergency surgical intervention.

During the operation, floppy lateral position was adopted for the exposure of fractures. First, we choose Kocher–Langenbeck approach to fully expose operative field. The femoral head was dislocated and consisted of larger head fragment and smaller articular fragments. Two Herbert screws countersunk below the articular surface were used to fix the fragments, after which there was still an osteochondral defect of the femoral head. The defects were covered with autogenous bone from the ilium. The femoral head was fixed on the acetabulum by a Kirschner wire in order to deal with femoral neck fracture continuously. Three hollow screws were used to repair femoral neck fracture after a satisfactory restoration. Then, we used 2 countersunk hollow screws and a reconstruction plate for reduction and fixation of fracture of the acetabulum posterior wall. Second, we select an ilioinguinal approach for repairing the transverse fracture of acetabular by a long pelvic reconstruction plate. At the end, we repaired the articular capsule and muscle tissue.

**Figure 2:**
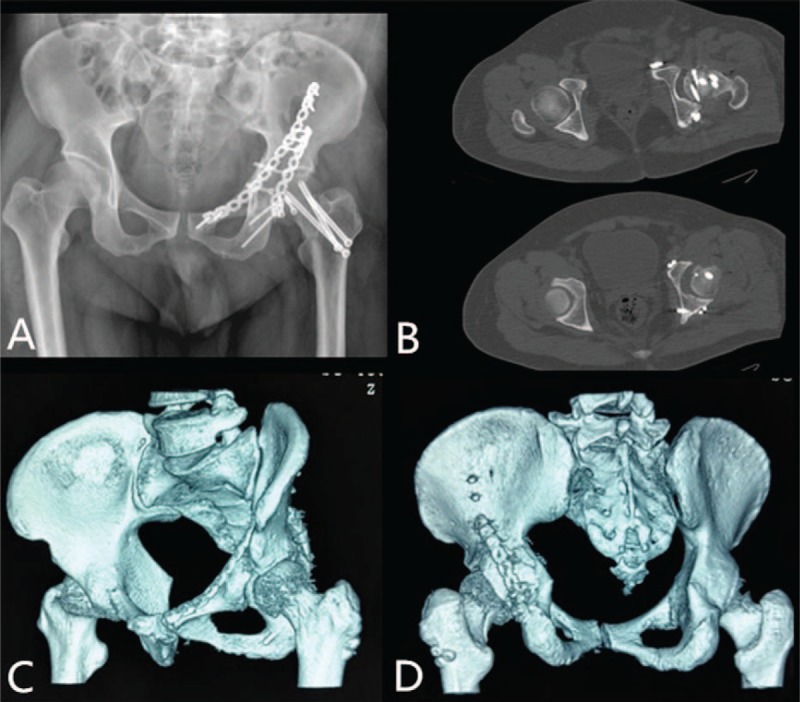
(A) Postoperative pelvic radiograph showing reduction of fractures. (B) Axial computed tomography showing no displacement of the femoral head or posterior wall. (C and D) Three-dimensional computed tomography showing good reductions of femoral head, femoral neck, and acetabular.

**Figure 3:**
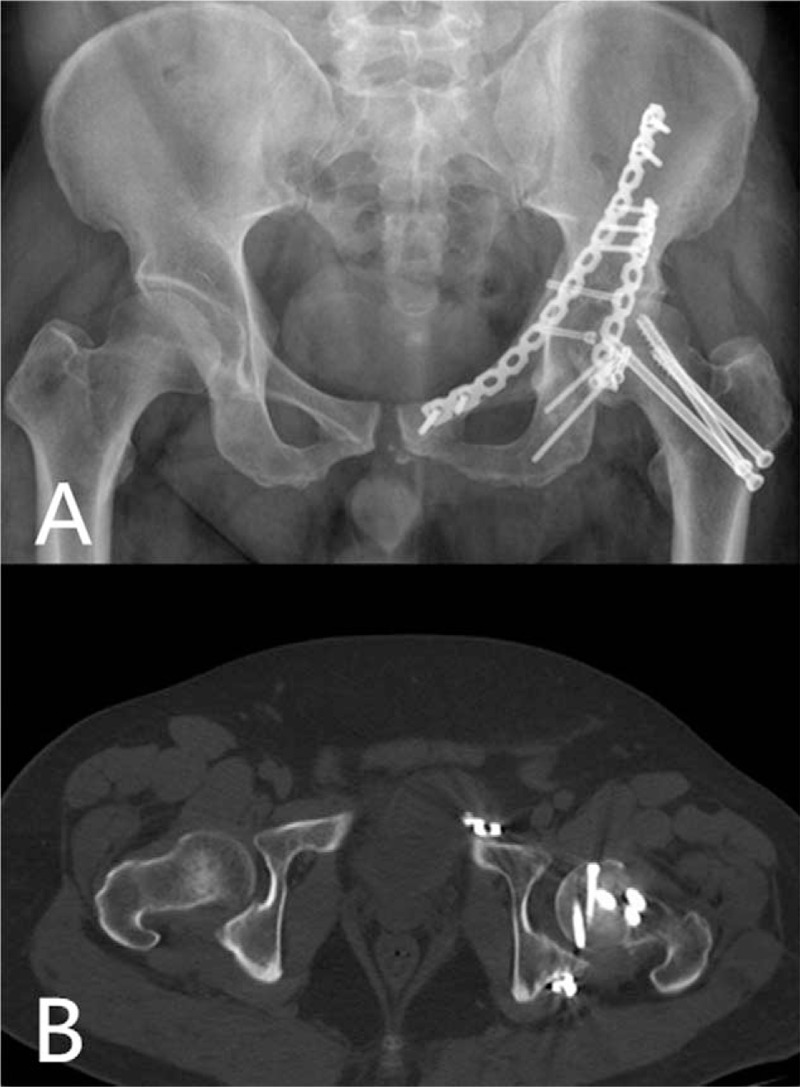
(A and B) Anteroposterior x-ray and CT images showing union of fractures with no femoral necrosis signs. CT = computed tomography.

Postoperative x-rays and CT scan of the hip demonstrated an ideal reductions of femoral head, femoral neck, and acetabular (Fig. 2A–D). The patient received a 14-day course of 750 mg/day of indometacin for heterotopic ossification prophylaxis. Rehabilitation was started after surgery, but weight bearing with a walker was allowed 8 weeks later. At 12-months follow-up, x-rays and CT showed satisfactory union of fractures (Fig. 3A and B), and the patient could walk freely and perform activities of daily living with no pain.

## 4 Discussion

Posterior dislocation of hip joint with femoral head fracture is not common.^[1]^ Epstein–Thomas type V fracture dislocation was divided into 4 subtypes by Pipkin,^[2,4]^ of which type III lesions are relative rare, caused by severe high energy injuries and characterized by fractures of the femoral head and neck.^[2,5]^ It is difficult to make surgical operation for type III fractures which often have poor prognosis.^[5,6]^ In addition to the fractures of the femoral head and neck, the case we treated was also associated with ipsilateral transverse-posterior wall acetabular fracture. To our knowledge, it is the first reported cases of such a complex fracture. The injuries are not appropriate for hip replacement because of transverse acetabular fracture, especially for young patients. The timing of the surgical treatment and the way in which surgery is performed are important.

Femoral head fracture is mainly seen in young adults, and the majority mechanism is due to dashboard injury in crushing vehicles.^[4]^ The mechanism of hip dislocation associated with fractures of the femoral head and acetabular fracture is very complicated.^[2,4]^ Acetabular fracture is due to external forces acting on the lateral trochanter of the femur, which is transmitted along the axis of the femoral neck.^[7]^ The femoral neck fracture is often caused by continued transmission of the force, which firstly makes the femoral head fracture.

Pipkin fracture is often due to serious traffic accidents. Therefore, we must verify whether there are other combined injuries, such as thoracic injury, abdominal injury, and traumatic brain injury, which may lead to life-threatening conditions.^[8]^ X-rays and CT scan evaluation were necessary to define the exact pattern of the fracture classification as soon as possible, which can help the surgeon to plan the operative approach.^[9]^ In our case, femoral head was blocked by the posterior margin of acetabulum because of severe comminuted fractures and hip dislocation. Closed reduction had no effect because of femoral neck fracture. Open reduction and internal fixation were performed as soon as possible to decrease pressure on femoral head articular cartilage and avoid femoral head osteonecrosis.

Femoral head can be easily fixed by the anterior approach in patients, but it is prone to avascular necrosis of the femoral head.^[10]^ We can repair the transverse acetabular fracture through the ilioinguinal approach, which is the most classic acetabular anterior approach.^[11]^ The K–L approach can reveal the entire posterior acetabular wall, and we can deal with fractures easily.^[12]^ It can be used for fractures of femoral head and acetabulum, and it could protect the anterior capsule. In our case, we performed a K–L approach combined with ilioinguinal approach. We should repair the fractures of femoral head and neck before acetabular, for manipulating and observing the femoral head easily.^[13]^ Intact femoral head could also be a sign of reduction of femoral neck fracture and acetabular fracture.

If the fracture fragment is large enough to allow internal fixation, we should attempt to make anatomical reduction of the femoral head fracture, which can make the femoral head articular surface smooth.^[14]^ We should use the countersunk head screw to avoid screw protruding into the joint. Herbert nail is a kind of titanium countersunk screw, which has the advantages of good compatibility, high fixation strength, easy operation, and no rejection reaction. In our case, we used 2 Herbert screws for the internal fixation of femoral head because of the advantage. Osteochondral loss located in anterior and inferior of the femoral head without weight bearing and we used the autogenous bone from the ilium which may promote fracture healing.

Many treatment options about femoral neck fracture have been described in the literature, such as cannulated screws fixation and prosthetic replacement. For the elderly with type III fractures, hip arthroplasty is the optimal method because the incidence rates of nonunion and avascular necrosis are very high. In contrast, the open reduction and internal fixation are considered for young patients.^[15]^ Three hollow screws were used to repair femoral neck fracture in our patient, who is a young man of 34-year-old with transverse acetabular fracture.

There was fracture displacement in the posterior wall of acetabulum, which needed internal fixation for reconstruction. We used 2 countersunk hollow screws and a reconstruction plate for anatomical reduction and fixation of fracture of the acetabulum posterior wall. Then we perfomed an anterior approach to repair the transverse acetabular fracture. Shaping plate was implanted and it allowed the patient early exercise.

The most common complications are nonunion, necrosis of femoral head, heterotopic ossification, traumatic osteoarthritis, and stiffness. Posterior dislocation of hip may happened again because of instability. We used indometacin for the prevention of heterotopic ossification. Early exercise was started after surgery for avoiding hip stiffness. At 12-months follow-up, the patient could walk freely and perform activities of daily living with no pain. Radiographic examination revealed fracture healing with no femoral necrosis signs.

## 5 Conclusion

We reported a rare case of Pipkin III fractures combined with complicated acetabular fracture in a 34-year-old male. This lesion is devastating and unrecoverable. Early surgical treatment is important. Appropriate approach and fixation could get satisfactory results with a long term follow-up.
